# ReLiance: a machine learning and literature-based prioritization of receptor—ligand pairings

**DOI:** 10.1093/bioinformatics/bts391

**Published:** 2012-09-03

**Authors:** Ernesto Iacucci, Léon-Charles Tranchevent, Dusan Popovic, Georgios A. Pavlopoulos, Bart De Moor, Reinhard Schneider, Yves Moreau

**Affiliations:** ^1^IBBT Future Health Department/ESAT-SCD, KU Leuven, Kasteelpark Arenberg 10, 3001, Heverlee-Leuven, Belgium; ^2^Luxembourg Centre for Systems Biomedicine (LCSB), University of Luxembourg, Campus Limpertsberg, 162 A, avenue de la Faïencerie, 1511 Luxembourg, Germany

## Abstract

**Motivation:** The prediction of receptor—ligand pairings is an important area of research as intercellular communications are mediated by the successful interaction of these key proteins. As the exhaustive assaying of receptor—ligand pairs is impractical, a computational approach to predict pairings is necessary. We propose a workflow to carry out this interaction prediction task, using a text mining approach in conjunction with a state of the art prediction method, as well as a widely accessible and comprehensive dataset.

Among several modern classifiers, random forests have been found to be the best at this prediction task. The training of this classifier was carried out using an experimentally validated dataset of Database of Ligand-Receptor Partners (DLRP) receptor—ligand pairs. New examples, co-cited with the training receptors and ligands, are then classified using the trained classifier. After applying our method, we find that we are able to successfully predict receptor—ligand pairs within the GPCR family with a balanced accuracy of 0.96. Upon further inspection, we find several supported interactions that were not present in the Database of Interacting Proteins (DIPdatabase).

We have measured the balanced accuracy of our method resulting in high quality predictions stored in the available database ReLiance.

**Availability:** http://homes.esat.kuleuven.be/~bioiuser/ReLianceDB/index.php

**Contact:** yves.moreau@esat.kuleuven.be; ernesto.iacucci@gmail.com

**Supplementary information:** Supplementary data are available at *Bioinformatics* online.

## 1 INTRODUCTION

The ‘omics’ era has presented tremendous opportunities for high-throughput investigations into important questions facing the research community. Many investigative strategies of implementing data mining techniques in combination with high throughput experiments have accomplished much. Several of these high-throughput experimental methods, yeast two-hybrid systems—Y2H (Ito *et al.*, 2001), pull-down assays (Vikis and Guan, 2004), tandem affinity purification (Puig *et al.*, 2001), mass spectrometry (Gavin *et al.*, 2002; Puig *et al*., 2001), microarrays (Stoll *et al.*, 2005) and phage display (Willats, 2002), have all generated enormous datasets, yet they are incomplete and are composed of many false positives and false negatives.

Several databases exist to store information about validated or predicted protein–protein interactions (PPI). They include the Munich Information Center for Protein Sequences—MIPS database (Mewes *et al*., 2004), the Molecular Interactions—MINT database (Zanzoni *et al*., 2002) the IntAct database (Kerrien *et al*., 2007), the Database of Interacting Proteins—DIP (Xenarios *et al.*, 2000), the Biomolecular Interaction Network Database—BIND (Bader *et al.*, 2001) and the BioGRID database (Stark *et al.*, 2006). Some, like the Yeast Proteome Database (Hodges *et al.*, 1999) contain interactions which are derived from wet lab results, as well as those curated from literary sources.

Currently, there exists thousands of candidate receptors and ligands and potentially hundreds of thousands of interactions. As the exhaustive assaying of every possible receptor—ligand pairs is impractical, a computational approach to the prediction task is necessary. For example, Gertz *et al.* (2003) created a receptor—ligand matching algorithm for the *chemokine* and *tgfβ* families. Later, we more aptly matched the *tgfβ* family with kernels (Gertz *et al.*, 2003; Iacucci *et al.*, 2011) with an increase in recall of 0.76 over the 0.44 obtained from the results of Gertz *et al.* Following this, we have benchmarked several machine learning techniques, and assayed several parameters, on the receptor—ligand interaction prediction task (Iacucci *et al.*, submitted for publication). The results of this work show that we can obtain a balanced accuracy of 0.84 in this prediction task.

Having used a ‘golden standard’ (Graeber and Eisenberg, 2001) to determine which is the best machine learning technique to apply to this problem, we now seek to make *in silico* predictions. The starting point of this novel course of research is the widely applied and powerful field of text mining. In this article, we present a strategy that takes into account text mining information in conjunction with a popular machine learning algorithm, the random forest (Qi *et al.*, 2005), which integrates several data sources such as domain, expression and phylogenetic-based evidence.

We build our new candidate list by searching for genes that are co-cited with the receptors and ligands from the DLRP database (Graeber and Eisenberg, 2001). We then make predictions using our trained classifier and evaluate the results in terms of known pairs, as well as in terms of distribution of the co-citations in our ranked list of predictions.

## 2 METHODS

### 2.1 Overview

**Fig. 1.**
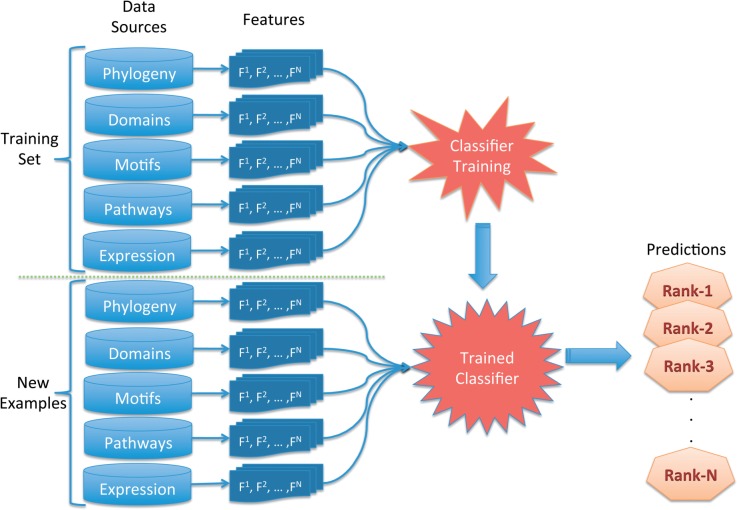
Family analysis. Candidates from the new examples where mapped to Gene Ontology and a search was performed for classifications containing the term ‘receptor’ with more than five members. The three classifications resulting from this search criteria were ‘Peptide Receptor Activity, G-protein Coupled’, ‘Transmembrane Receptor Tyrosine Kinase Activity’ and ‘Cytokine Receptor Activity’. We then used the DIP database as a baseline for calculations of sensitivity, specificity and balanced accuracy for each classification

We propose a pertinent process (Fig. 1) to carry out this prediction task, using a state-of-the-art prediction method and an accessible and comprehensive dataset. Among several classifiers, random forests have previously been identified to work best for this prediction task (Iacucci *et al.*, submitted for publication). The training of this classifier was carried out using an experimentally validated dataset of receptor—ligand pairs (Graeber and Eisenberg, 2001). New examples are then classified using the trained classifier. The process begins with the collection of candidate genes to enter as new examples to our trained classifier. The new examples may be derived in a variety of ways (an up-regulated gene list, genes that contain a specific protein domain, protein-array experimentation). In our setting, we look at genes that are co-cited with the receptors and ligands from the DLRP database. The DLRP was constructed using experimentally determined ligand-receptor cognate pairs through a literature review. The database contains 314 proteins, 210 of which are used in our training set.

### 2.2 Creation of the candidate list

We created a candidate list by taking the 210 receptors and the ligands in the DLRP database (Graeber and Eisenberg, 2001) and finding all the co-cited genes [using the text mining track of the STRING database (Von Mering *et al.*, 2007)] for which all the information from all the data sources was available. In total, 483 candidates form this list.

### 2.3 Data sources

The data were collected and processed as reported in Iacucci *et al.* (submitted for publication). Briefly, profiles associated to genes were retrieved from various databases [i.e. Kegg, Interpro, Toucan, (Aerts *et al.*, 2005; Hunter *et al.*, 2009)]. These profiles are vectors of measurements, which represent the candidate ligand or receptor. We create a feature value for each candidate receptor–ligand pair by taking the two vectors and applying a pairwise similarity measure (cosine, mutual information, absolute correlation coefficient, jaccardi coefficient) to them. This value is now the feature value that corresponds to the similarity between each ligand and receptor. The feature values which arise from the use of the various sources are then inputted into our random forest.

These profiles contained between 79 (expression data) and 674 features (domain data) depending on the data source being considered. For the phylogenetic vector, complete protein sequences were retrieved for seven species (*Rattus norvegicus*, *Mus musculus*, *Homo sapiens*, *Pan troglodytes*, *Canis familiaris*, *Cavia porcellus* and *Bos taurus*) from EnsEMBL build 51 (Hubbard *et al.*, 2009). Sequences were then aligned using ClustalW (Thompson *et al.*, 1994) to detect orthology. The gene expression profiles were retrieved from the Genomics Institute of the Novartis Research Foundation (GNF) human expression atlas (Su *et al.*, 2004). Each profile contains 79 values corresponding to the 79 conditions considered by (Su *et al.*, 2004). The domain information is retrieved from the InterPro database (Hunter *et al.*, 2009), through EnsEMBL. Only the domains present in at least one of the 210 receptors and ligands considered are kept for further analysis. The motif data was created using the Toucan toolbox to search for putative motifs in the upstream sequence of the genes. Each protein is then represented by a vector of size 674. Each value represents the score of the corresponding motif for the given protein. The pathway data were retrieved from the Kegg Pathway database (Aerts *et al.*, 2005). Only the pathways in which at least one of the 210 training example receptors and ligands is involved are used to build the final profiles. This means that, in the case of Kegg, candidate proteins are represented by sparse binary vectors of size 314.

### 2.4 Classifier

Following our previous work, we selected the method that provides the best balanced accuracy (Iacucci *et al.*, submitted for publication). This method was found to be the random forest with a balanced accuracy of 0.84 (Iacucci *et al.*, submitted for publication). We used the best performing similarly measures for each data source (domain, phylogenetic—absolute correlation coefficient; expression, kegg, motif—absolute cosine). Our classifier was trained using the DLRP dataset described previously (Graeber and Eisenberg, 2001). We applied Matlab 2010a implementation of random forest (class TreeBagger) with the number of trees set to 1000 and the number of variables to select at random for each decision split set to default value (square root of number of variables). To achieve stability in the prioritized lists, the algorithm has been trained and tested 200 times, after which the new predictions were assigned by averaging scores. The running time for this procedure was ~2h.

The candidate list was inputted into the trained classifier (trained and calibrated as described above). Each possible receptor—ligand pair was classified as interacting or noninteracting and was ranked according to a score assigned by algorithm—that is, the probability of observation belonging to particular class given as fraction of observations of that class in predicted leafs across the ensemble. The resulting list is of size 116 403 with 7521 positive predictions, 108 882 negative predictions and 7958 predictions for which co-citations exist.

### 2.5 Co-citation and receptor family analysis

Co-citation values were downloaded from text mining track of the STRING database (Von Mering *et al.*, 2007). The normalized co-citation score is the total number of co-citations for the members of a bin divided by the connectivity score for the members of a bin and then scaled to the maximum value across all bins. The co-citation analysis was performed by comparison of the ranked results with the co-citation score. In order to assess the biological relevance of our work in terms of individual receptor families, we map our candidates to Gene Ontology (GO) classifications and examine the performance of our predictions to individual receptor families. Gene Ontology analysis was carried out by mapping the candidate list to gene ontology classifications that contained the word ‘receptor’ and had more than five members. Three classifications resulted from this search criteria (‘Peptide Receptor Activity, G-protein Coupled’, ‘Transmembrane Receptor Tyrosine Kinase Activity’ and ‘Cytokine Receptor Activity’). The predictions from our method were then assessed using the members of each of these classifications by comparing our predictions with the interactions reported in the DIP (Xenarios *et al.*, 2000) database for these classifications as it is known to contain experimentally validated receptor—ligand pairings (Graeber and Eisenberg, 2001).

## 3 RESULTS AND DISCUSSION

Evaluating the results of our predictions can be challenging, as the overall objective of our work is to predict novel receptor—ligand pairs, yet the merits of this work must be measured using known interactions. In order to address this, we look at the qualitative aspect of the co-citation profile of the ranked predictions. Furthermore, we look at the receptor classifications of Gene Ontology and assess our ability to make predictions in terms of sensitivity, specificity and balanced accuracy with respect to the known DIP interactions for these families. Finally, we assess the predictions one-by-one by examining the top ten predictions in the different quarters of the ranked predictions.

### 3.1 Co-citation analysis

**Fig. 2.**
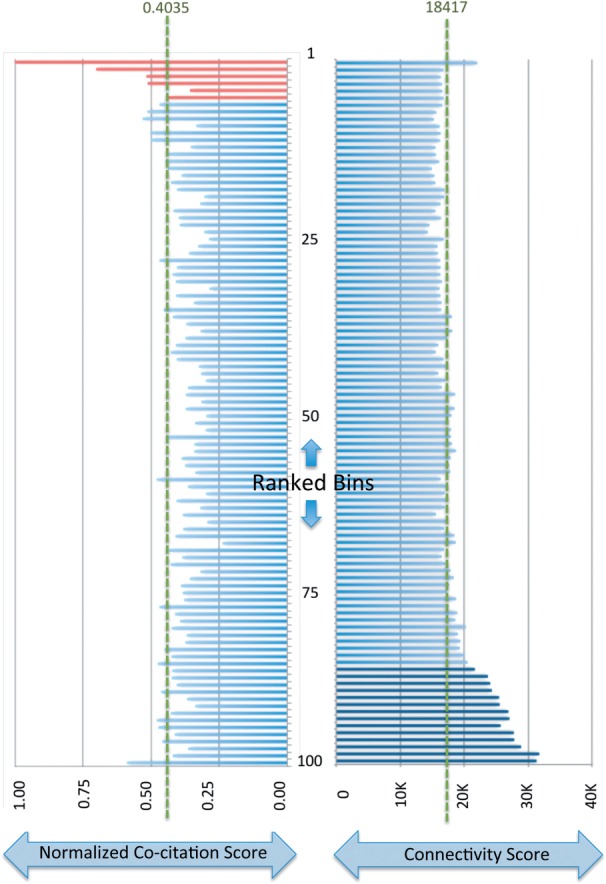
Histogram. The prioritized list resulting from our workflow is binned into 100 ranked bins of size 1164. On the left we see the ranked bins that have a length corresponding to their normalized co-citation score. The normalized co-citation score is the total number of co-citations for the members of a bin divided by the connectivity score for the members of a bin and then scaled to the maximum value across all bins. The bins colored in red correspond to the bins that contain pairs, which are called a positive by our classifier. On the right, we see the ranked bins that have a length corresponding to their connectivity score. The connectivity score is the total number of edge degree (number of predicted interactors in the genome) of each of the members of the bin. The dark blue bins correspond to bins, which contain members with higher connectivity than average. The green dashed lines correspond to the average value across all bins

Assuming co-citation should positively correlate with correct pairing of candidate pairs, we examine the predicted prioritized candidate list in terms of co-citation. The overall shape of the prioritized candidate list (Fig. 2) suggests that the performance of our workflow is consistent with an accurate classification strategy. Looking at Figure 2, we see the 100 ranked bins containing the co-citation values for the prioritized list. Looking at the top six bins, we find that there is a peak in co-citation, suggesting an overall positive prediction area. Indeed, if we examine the first quarter of the bins, we see that there is an enrichment of co-cited terms as they are higher than the average level (value 0.4035), which one would expect to see if the co-citations were uniformly distributed (*P*-value <0.05 when applying a *t*-test between the first and second quarters). Looking at the second and third quarters, we see a depletion of co-cited terms in those bins, whereas the fourth quarter contains levels of co-citation close to the average level. This plot suggests that our classifier is able to learn the structure in the data of interacting receptors and ligands, as well as noninteracting pairs, thus explaining the enriched and depleted areas of the plot (*P*-value <0.05 when applying a *t*-test between the first and second quarters). The bins toward the end of the ranking, which measured at about the average level, suggest that a low classifier score is assigned to pairs that show structure related to neither interacting nor noninteracting pairs. In fact, we examined these pairs and found that they were highly connected relative to the rest of the list, suggesting that the classifier could better identify pairs with where the candidate ligand and receptor had fewer, specific interactions and not more promiscuous proteins.

### 3.2 Receptor Family analysis

**Fig. 3.**
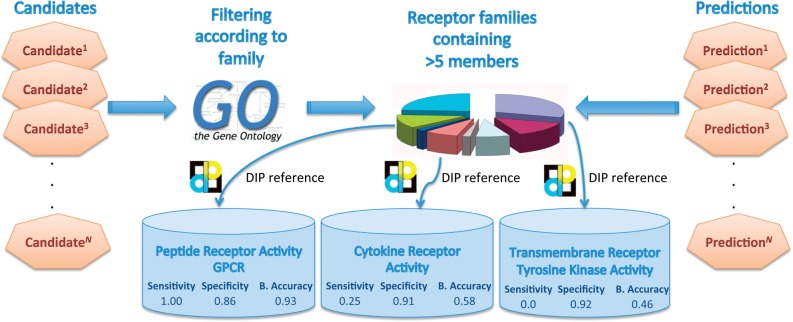
Workflow. The trained classifier is provided with new examples (genes which are co-cited with the receptors and ligands from the DLRP database) and makes predictions based on its ability to distinguish between interacting and noninteracting pairs. The predictions are ranked by the random forest score provided by the class

In order to find areas of high competency in our prioritization, we map the candidates to Gene Ontology [using DAVID (Dennis, Jr. *et al.*, 2003)] and perform a search for classifications containing the term ‘receptor’ and found three classifications with more than five members (Fig. 3). The three classifications resulting from this search criteria were ‘Peptide Receptor Activity, G-protein Coupled’, ‘Transmembrane Receptor Tyrosine Kinase Activity’ and ‘Cytokine Receptor Activity’. We then used the DIP database as a baseline for calculations of sensitivity, specificity and balanced accuracy for each classification. More specifically, we take the candidates from our experiment that are mapped to these classifications and compare our positive predictions with those reported in the DIP database. We find that we are able to successfully predict receptor—ligand pairs within the G-protein coupled receptors (GPCR) family with a balanced accuracy of 0.96. The GPCR family represents the most important group of current drug targets because 40% of all modern medicinal drugs are GPCR related (Overington *et al.*, 2006) (e.g. imatinib, cetirizine, hydroxyzine and acebutolol). This is no surprise as they are key agents in several diseases (Overington *et al.*, 2006) (e.g. WHIM syndrome, Retinitis pigmentosa and Cryptorchidism). We searched our results for novel interactions that were made with members of this family, which did not exist in the DIP database. We examined the top 10 predictions (Table 1) from our prioritization involving members of the GPCR family found several supported interactions such as those between TCF7-CTNNB1 (Kerrien *et al.*, 2007; Mewes *et al.*, 2004; Xia *et al.*, 2006), LEF1-CTNNB1 (Bader *et al.*, 2001; Goel *et al.*, 2012; Mewes *et al.*, 2004; Stark *et al.*, 2006) and ANGPT2-F2R (Kerrien *et al.*, 2007). In addition, we find high STRING (Von Mering *et al.*, 2007) scores for the following interactions CCL22-CCR1, CXCL13-CCR1 and CCL22-CX3CR1. Notably, we found that two of these novel *in silico* predictions (CXCL13-CCR1 and CCL22-CX3CR1) showed experimental evidence of interaction (Booth *et al.*, 2008; Hoglund *et al.*, 2011).

**Table 1.** *In Silico* GPCR predictions: top ten predictions made in the GPCR family of receptors and ligands

*In Silico* GPCR Predictions				
**Rank**	**Query protein**	**Predicted partner**	**Score**	**Evidence of interaction**
1	CD27	CX3CR1	0.789	
2	TCF7	CTNNB1	0.780	INTNETDB, MIPS, INTACT
3	CD27	CCR1	0.723	
4	CCL22	CCR1	0.721	High STRING prediction: 0.964
5	LEF1	CTNNB1	0.720	BIND, BIOGRID, HPRD, MIPS
6	CCR1	CSF1	0.716	
7	CXCL13	CCR1	0.694	High STRING prediction: 0.983 Experimental (Booth *et al.*, 2008)
8	EDAR	CX3CR1	0.680	
9	ANGPT2	F2R	0.667	INTACT
10	CCL22	CX3CR1	0.638	High STRING prediction: 0.945 experimental (Hoglund *et al*., 2011)

### 3.3 Qualitative analysis

We examine the 10 co-cited predictions made at the top of each quarter of our ranking and find that the several of the predictions made at the top of the 1st quarter were supported (Table 2). Among these interacts are those between CD3G-CD3D (Goel *et al.*, 2012; Kerrien *et al.*, 2007; Mewes *et al*., 2004; Stark *et al.*, 2006), B2M-CALR (Goel *et al.*, 2012; Kerrien *et al.*, 2007; Mewes *et al.*, 2004; Stark *et al.*, 2006), CDC14A-CDC7 (Kerrien *et al.*, 2007), PDGFRB-GRB7 (Goel *et al.*, 2012; Mewes *et al.*, 2004) and SMAD7-ACVRL1 (Kerrien *et al.*, 2007). The second and third quarters (which were below the threshold of a positive call in the algorithm) contain predictions that were not supported. The remaining, fourth quarter contained three supported interactions (Supplementary Tables 1, 2 and 3). The interactions present in the fourth quarter are due to the highly connected nature of the interacting receptors and ligands that are assigned low scores (as described above).

**Table 2.** Top 10 co-cited predictions: the top 10 co-cited predictions with co-citation

**TOP 10, first quarter**				
**Rank**	**Query protein**	**Predicted partner**	**Score**	**Evidence of interaction**
1	CD3G	CD3D	0.903	BIOGRID, HPRD, INTACT, MIPS
2	CRK	ALK	0.894	
3	AC003958.6.1	TNFSF4	0.890	
4	B2M	CALR	0.879	BIOGRID, HPRD, MIPS
5	CDC14A	CDC7	0.876	INTACT
6	DCN	SMAD7	0.870	
7	PDGFRB	GRB7	0.867	HPRD, MIPS
8	SMAD7	ACVRL1	0.866	INTACT
9	TNFSF4	IL18	0.866	
10	WDR48	ERBB2	0.865	

### 3.4 ReLiance database

The ReLiance database is available at: http://homes.esat.kuleuven.be/~bioiuser/ReLianceDB/index.php. Among the information accessible from the database are the putative protein partner, the score for our prediction, as well as other pertinent information (hugo names, swissprot ids, PDB entries, etc.). The database entries are annotated and enriched on-the-fly using the reflect API (Pafilis *et al.*, 2009).

A mouse-click function is applied to the highlighted proteins and genes to generate informative pop-up windows hosting information where the specific bioentities from public databases are summarized. Thus, links to the synonyms, the complete sequence of the longest transcript, domains from the Simple Modular Architecture Research Foundation (SMART) (Letunic *et al.*, 2006) database, the PDBsum (Laskowski, 2001) structure, the interactions from STITCH (Kuhn *et al.*, 2008), the known subcellular location and the source organism are shown, respectively. Most of these features on the pop-up are hyperlinked to related database entries.

In addition, Medline abstracts concerning the bioentity are offered by the iHOP (Hoffmann and Valencia, 2004) service. Similar functionality can be offered by OnTheFly (Pavlopoulos *et al.*, 2009) service for a more targeted search as selected results can be stored locally and then annotated. This way, in terms of data integration and identifier updates, the database can always be up to date and supported by the Reflects and OnThFly's dictionary.

Networks showing the interaction partners can dynamically be explored by the Medusa application (Pavlopoulos *et al.*, 2011) that runs as an applet on our site. Force-directed layout algorithms provide intuitive layouts, whereas the color scheme encodes certain information. The ‘purple’ node represents the query protein, the ‘red’ nodes correspond to the predicted partner proteins which are also co-cited to the query protein and ‘yellow’ nodes correspond to the predicted partner proteins that are not co-cited with the query protein.

## 4 CONCLUSIONS AND FUTURE DIRECTIONS

Our workflow to carry out the receptor—ligand pairing prediction task provides several findings. Using a text mining approach in conjunction with a state of the art prediction method, as well as a widely accessible and comprehensive dataset, we have produced a prioritized list that is consistent with a successful classification scenario. In addition, we find that we are able to successfully predict receptor—ligand pairs within the GPCR family with a balanced accuracy of 0.96. We introduce ReLiance, our database for predicted receptor—ligand pairings which provides high data integration and visualization capabilities.

*Funding:* This work was supported by Research Council KUL (ProMeta, GOA Ambiorics, GOA MaNet CoE EF/05/007); SymBioSys en KUL PFV/10/016 SymBioSys; START 1 (several PhD/postdoctorate and fellow grants); Flemish Government: FWO: [PhD/postdoctorate grants, projects G.0318.05 (subfunctionalization), G.0553.06 (VitamineD), G.0302.07 (SVM/Kernel), research communities (ICCoS, ANMMM, MLDM); G.0733.09 (3UTR)]; G.082409 (EGFR) IWT: PhD Grants, Silicos; SBO-BioFrame, SBO-MoKa, TBM-IOTA3 FOD: Cancer plans, IBBT; Belgian Federal Science Policy Office: IUAP P6/25 (BioMaGNet, Bioinformatics and Modeling: from Genomes to Networks, 2007- 2011); EU-RTD: ERNSI: European Research Network on System Identification; FP7-HEALTH; CHeartED

*Conflict of Interest:* None declared.
